# Supplementary material for: Atomic vapor as a source of tunable, non-Gaussian self-reconstructing optical modes
Source: Sci Rep. 2017 Feb 8;7:42311. doi: 10.1038/srep42311 (PMC5296730; doi:10.1038/srep42311)
Supplement: Supplementary Information [file srep42311-s1.pdf]

# Supplementary Information:
# Atomic vapor as a source of tunable, non-Gaussian self-reconstructing optical modes

**Jon D. Swaim[1], Kaitlyn N. David[1], Erin M. Knutson[1], Christian Rios[1], Onur Danaci[1], and Ryan T. Glasser[1,*]**

[1]Department of Physics, Tulane University, New Orleans, LA USA 70118
[*]rglasser@tulane.edu

## ABSTRACT

In this supplemental document, we include experimental results and details of the calculations supporting the results shown in the manuscript. In particular, we have included data on the frequency dependence of the mode shapes; a series of full images recorded during our study of self-healing; and an additional calculation on image correlations between the reference and unobstructed propagating modes.

## Frequency dependence of non-Gaussian mode shapes

In Fig. 2 of the manuscript, we demonstrated that the generation of non-Gaussian modes can be tuned by varying the input optical power and the vapor cell temperature. In addition, we observed that a similar effect can be achieved by tuning the laser frequency. In the experiments, the laser frequency was locked to a fixed detuning from the resonance (see Methods section in the manuscript), to prevent fluctuations and/or drift in the laser frequency from altering the shape of the optical modes. In principle, adjusting the laser frequency could enable further tunability. In Fig. S1 (a), we show a smoothed two-dimensional intensity projection of cross-sections obtained from images of the modes for various detunings. The dashed lines indicate the detunings of -80 MHz and -275 MHz used for the experiments in the manuscript. The cross-sections for those detunings are shown in Fig. S1 (b).

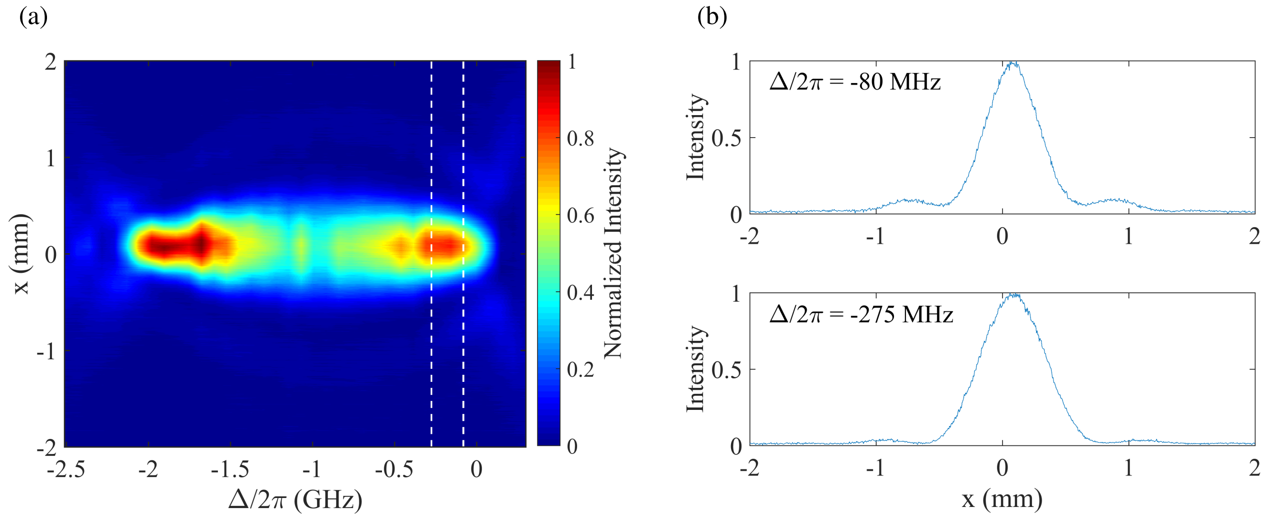


**Figure S1.** **Frequency dependence of non-Gaussian mode shapes.** **(a)** Smoothed intensity projection of cross-sections obtained from images of the generated non-Gaussian modes at various detunings. $P \sim 250$ mW. $T \sim 125°$C. **(b)** Corresponding cross-sections for the modes at detunings of -80 MHz and -275 MHz.

## A study on self-healing

In Fig. 4 of the manuscript, we show that the optical beams exhibit the ability to reconstruct after encountering an obstacle. We compare the spatial structure of the reconstructing modes with a reference (the unobstructed mode at $z = 0$ m) using the two-dimensional correlation function. In general, the correlation function for two images $A_{mn}$ and $B_{mn}$ is

$$\eta = \frac{\sum_m \sum_n (A_{mn} - \bar{A}) \cdot (B_{mn} - \bar{B})}{\sqrt{\left(\sum_m \sum_n (A_{mn} - \bar{A})^2\right) \cdot \left(\sum_m \sum_n (B_{mn} - \bar{B})^2\right)}} \tag{1}$$

where $A_{mn}$ and $B_{mn}$ are the intensities recorded by the CCD camera, and $\bar{A}$ and $\bar{B}$ represent their mean values. In calculating the correlation between the reconstructed modes and a reference at the origin (Fig. 4 (c) of the manuscript), we made two separate calculations: (1) using the area of the entire image, and (2) using only the area covered by the obstruction. In the manuscript, we focus on the latter case and show a high degree of correlation for the reconstructed mode at $z \sim 7.5$ m. For completeness, in Fig. S2 we include a sample of some entire images, which illustrate both the regeneration of the mode within the obstruction area as well as the evolution of the mode (outside that area) as a function of the propagation coordinate $z$. These images were used in calculating the former case. The result in this case, also shown in Fig. 4 (c) of the manuscript, is that the correlation decreases as a function of $z$. However, we do observe a slight increase in correlation around $z = 8$ m, in agreement with the calculation taken only over the obstruction area.

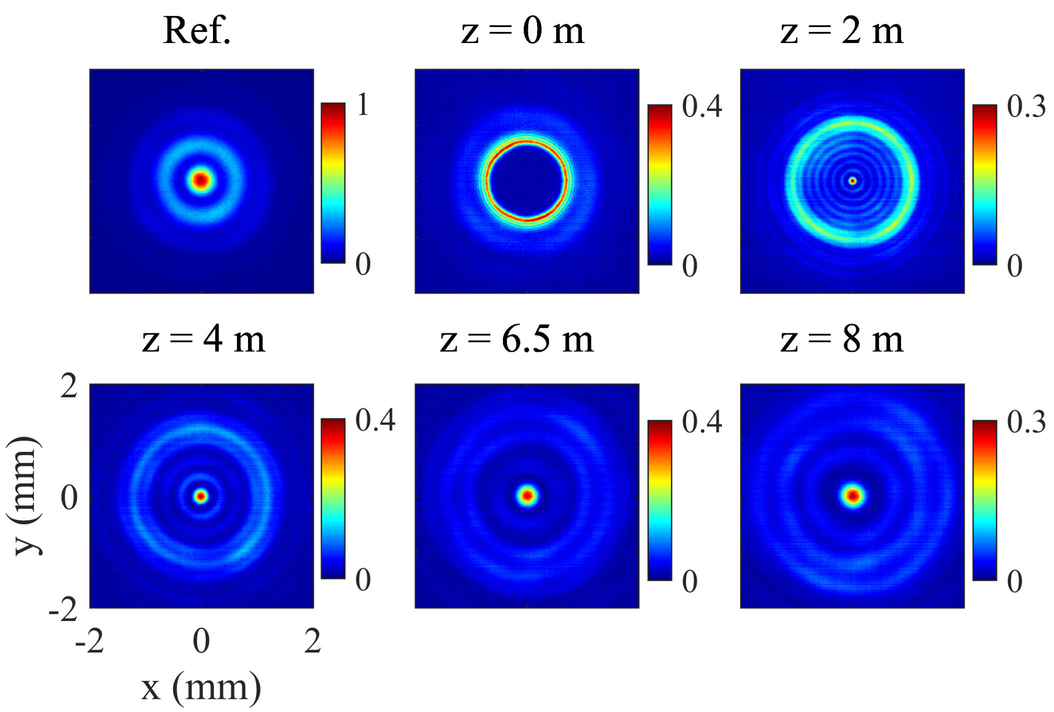


**Figure S2. Self-reconstruction of non-Gaussian modes.** Full single-shot images recorded on the CCD camera with an input power of $P \sim 300$ mW and a vapor cell temperature of $T \sim 125$°C. A circularly symmetric obstruction 3 mm in diameter blocks approximately 20% of the light at $z = 0$ m. The observed obstruction size is smaller than 3 mm, due to an additonal lens placed in front of the camera on account of the large size of the optical modes. Each image is first normalized with respect to its maximum for visual clarity, and then subsequently normalized with respect to the (unobstructed) reference at the origin.

Lastly, in Fig. S3, we show a calculation (via Eq. 1) of the correlation between the original reference mode at $z = 0$ m and the unobstructed propagating mode at various $z$. This calculation is taken for the entire images.

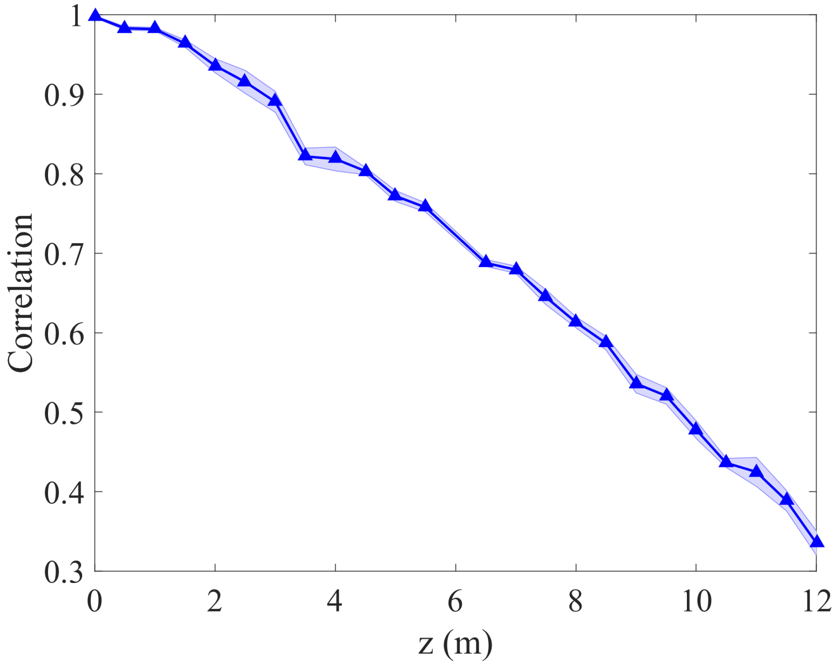


**Figure S3. Two-dimensional image correlation between the reference and unobstructed propagating modes.** The correlation is calculated for the entire image, and the shaded area indicates uncertainty based on one standard deviation.
